# Analysis of FIFA 2023 Women’s World Cup match performance according to match outcome and phase of the tournament

**DOI:** 10.5114/biolsport.2025.142643

**Published:** 2024-09-06

**Authors:** José M. Oliva-Lozano, Farzad Yousefian, Paweł Chmura, Tim J. Gabbett, Rick Cost

**Affiliations:** 1 United States Soccer Federation. Chicago, IL, United States; 2 CIDESD, Research Center in Sports Sciences, Health Sciences and Human Development, Department of Sport Sciences, University of Beira Interior, Covilhã, Portugal; 3 Department of Team Games, Wroclaw University of Health and Sport Sciences. Wrocław, Poland; 4 Gabbett Performance Solutions, Brisbane, QLD, Australia

**Keywords:** Female, Football, Professional, Elite, Competition

## Abstract

The aim of the study was to analyze the FIFA 2023 Women’s World Cup match performance according to match outcome and phase of the tournament. Match performance data were analyzed from a total of 64 games, which included a total of 32 national teams. The variables collected from the post-match summary reports were calculated according to match time (i.e., data per minute) and were categorized in four groups: key statistics, in possession and out of possession variables, out of possession defensive pressure variables, and physical performance variables. When it comes to match outcome, significant differences were observed in key statistics (e.g., there were significantly greater possession, goals, attempts at goal on target, total passes, total passes completed, defensive line breaks, and receptions in the final third when winning compared to drawing or losing), in possession variables (e.g., wins showed significantly greater build up unopposed compared to losses and draws), out of possession variables (e.g., wins showed significantly greater high press than losses), and out of possession defensive pressure variables (e.g., losses showed significantly greater total pressure, pressing direction outside, and ball recovery time compared to wins or draws). There were no significant differences in any physical performance variable, except for distance covered in zone 1 (losses > draws). When it comes to the phase of the tournament, no significant differences were observed in any variable, except for distance covered in zone 1 (knock-out > groups). Furthermore, significant interactions were observed between match outcome and phase of the tournament in the following variables: attempts at goal on target, pass completion %, progressions, and average pressure duration. Quantifying and comprehending match-play characteristics hold significant importance in guiding practices within women’s soccer. Therefore, this study serves as a resource for the staff of national teams to understand performance according to match outcome and phase of the tournament in an international women’s soccer tournament.

## INTRODUCTION

Interest in women’s soccer has grown exponentially, especially in the last decade [1, 2]. During this period, participation has increased by a third, and FIFA estimates that the number of female practitioners will reach 60 million in 2026 [3]. A recent study analyzed twenty years of the FIFA Women’s World Cup and found that elite women’s soccer has developed with regard to competitiveness, approaching the status already achieved by men [4]. This fast progress represents new challenges for the sports sciences and football associations. There has been a continuous growth in research attention [2]. For example, a recent scoping review on the physical demands of women’s soccer showed that it is the most researched sport in comparison with other elite team sports [1]. However, there is a great disparity in the volume of publications involving men and women, with fewer than 15% of all the studies carried out on professional soccer having female participants [5]. Despite the appearance of an increasing number of publications about professional female soccer players, the literature on match performance determinants is still insufficient, so it is necessary to have more gender-specific evidence.

The growth that women’s soccer is experiencing in terms of the number of players and economic investment [4] has led to greater professionalization over a short period of time. For example, the FIFA 2023 Women’s World Cup was observed to be a more competitive tournament compared to the editions held in 2015 and 2019 [6]. The physicality of women’s soccer has significantly intensified over the past decade, notably in terms of the speed and intensity of matchplay [7]. In addition, the impact of contextual factors in women’s soccer, such as home advantage or match status [8] has been the subject of research that has aided in the understanding of both individual and group behavior depending on various match situations and settings. However, so far, the impact of variables such as match outcome and phase of the tournament, except in individual cases, has been overlooked, particularly when considering the top teams competing in the World Cup.

Several notational analysis studies examining men’s soccer have shown that winning teams performed better in key performance variables such as ball possession, total passes, accurate passes, dribbles, crosses, corners, and goal-scoring opportunities (e.g., shots, shots on target) compared to losing teams [9, 10]. When examining the relationship between physical performance and match outcome in male soccer players, there is a consensus that players covered more highspeed and sprint distance when games are lost, while covering less distance at low and moderate speeds when games are won [11]. In the FIFA 2019 Women’s World Cup in France [12], winning teams had more possession of the ball, more shots, and more passes. Findings from another study using the 2019 World Cup data found that winning teams demonstrated a superior ability to maintain ball possession, particularly in the opponent’s half [13]. They also had a higher number of ball possessions during the early periods of both the first and second halves [13]. In addition, a different study, which used Football Association Women’s Super League, National Women’s Soccer League, European Cups, and World Cups data, found that successful teams had more effective ball movement and successful passes over the course of a season or tournament, with a more centralized distribution of ball possession [14]. Moreover, another study analyzed the influence of variables related to passes executed in the National Women’s Super League in the United States and the Football Association Women’s Super League in England, noting that winning teams exhibited, among other characteristics, a higher number of total passes and successful passes in the final third [15]. In turn, Trewin et al. claim that activity profiles, and more specifically, pacing strategies in female soccer, differ across teams and are dependent on game outcome and the quality of opposition players [16]. Such research is needed, as match performance variables are a valid means of assessing performance quality [17].

No significant studies have taken into account the phase of the tournament in their analyses. Previous attempts have been made to describe physical variables in successive rounds of the World Cup tournament in males [18, 19], but the effect of tournament phase on such a wide range of variables corresponding to match performance has not been strictly studied. Regarding analysis of female soccer players, a recent study indicated that specific fatigue patterns observed within and between matches throughout the Women’s World Cup may be influenced beyond playing positions, the rank of the opposition, as well as the stage of the tournament [20]. Given that research on technical, tactical, and physical indicators in women’s soccer dates back only a dozen years, it is important to provide updated analysis on this aspect.

To the best of our knowledge, limited research in women’s soccer research has analyzed both technical-tactical and physical performance data in relation to match outcome and tournament phase [21–23]. The aim of the study was to analyze the FIFA 2023 Women’s World Cup match performance according to match outcome and phase of the tournament.

## MATERIALS AND METHODS

### Study design

An observational study was conducted using post-match summary reports provided by FIFA during the 2023 Women’s World Cup in Australia and New Zealand. The FIFA post-match summary reports were downloaded at the end of the World Cup [24] to create a dataset, which consisted of match-related performance variables. Differences in match performance by match outcome and phase of the tournament were analyzed. Since these data were openly accessible, no ethical approval was required.

### Sample

Match performance of elite female soccer players was analyzed from a total of 64 games, which included 32 national teams. Participating teams were distributed into a total of 8 groups consisting of 4 teams. Each team played 3 matches in the group phase while the two higher-ranked teams from each group qualified for the knock-out stage. Since only qualifying teams moved to the round of 16 and only the winner of each match in each round moved to the next round (e.g., quarter-finals, semi-finals, play-off for third place, and final), the number of match observations by team was different (range: 3–7). Specifically, during the knock-out phase, if matches were still tied after a 30-minute extra time (which were included too), teams would go into a penalty kick shootout to determine the winner. However, the match outcome considered, for the purposes of this study, was the one right after the 30-minute extra time period (i.e., not the outcome after the penalty kick shootout), so each of the teams were assigned ‘draw’ as their final match outcome. This resulted in 128 team observations. The data were subsequently grouped by match outcome (win: W; draw: D; loss: L) and phase of tournament (group or knock-out phase). This generated a dataset of 96 groupphase matches (W – 38 observations; L – 38, and D – 20) and 32 knock-out phase matches (W – 12, L – 12, and D – 8).

### Procedures

A customed worksheet was created to analyze each teams’ and players’ match performance data. These data, which were available in the FIFA post-match summary reports [24], were collected by a multi-camera optical tracking system (TRACAB, ChyronHego, Sweden) and the player movements were recorded by high-definition cameras which operated at 25 Hz. The validity of the tracking system has been verified by previous research assessing the quality of data for soccer-specific performance analyses [25]. After system calibration and quality control procedures, the captured data were analyzed FIFA’s provider using match analysis software. The variables collected from the post-match summary reports were calculated according to the match duration (i.e., data per minute) and were categorized in four groups: key statistics, in possession and out of possession variables, out of possession defensive pressure variables, and physical performance variables. Detailed definition of the variables is presented in Table 1 [26–28].

**TABLE 1:** Definition of variables.

Variables	Definition
% Possession	Percentage of total time a team retains possession of the ball.

Goals	The total number of goals scored.

Attempts at Goal	The total number of actions performed with the intention of scoring a goal.

Attempts at Goal – On Target	The total number of actions performed as shots that are directed towards the goal and require intervention by the goalkeeper to prevent a goal, or that go into the goal themselves.

% Attempts at Goal – On Target	Percentage of the total number of on target attempts at goal divided by the total number of attempts to score a goal.

Total Passes	Total number of attempted distribution actions (pass) to keep possession of the ball.

Total Passes – Complete	Total number of completed distribution actions (pass) to keep possession of the ball.

% Pass Completion	The total number of completed passes divided by the total number of passes.

Completed Line Breaks	The total number of actions to progress the ball and break one more unit lines of the opposition team shape while maintaining ball possession.

Defensive Line Breaks	Total number of actions to progress the ball and break the opposition defensive line by playing the ball beyond the deepest player in the defensive line while maintaining possession.

Receptions in the Final Third	Total number of actions where the ball is received in the final third of the opposition team.

Crosses	The total number of actions performed where any ball is sent into the opposition team’s area from a wide position.

Ball Progressions	The total number of actions performed with the intention of breaching the opposition team shape while in possession of the ball.

Defensive Pressures Applied	The total number of defensive pressure actions performed to close down space on an opposition player in possession of the ball.

Defensive Pressures Applied – Direct Pressures	The total number of defensive pressure actions performed to close down space on an opposition player in possession of the ball that is close enough to the opposition play to be able to tackle/challenge/physically contest the ball and opposition action.

% Defensive Pressures Applied – Direct Pressures	The total number of direct defensive pressures applied divided by the total number of defensive pressures applied.

Forced Turnovers	The total number of actions performed to force opposition to lose possession of the ball and for the team to regain ball possession on the next touch through a misplaced/intercepted pass, a successful tackle or a lost dribble/ball carry.

Second Balls	The total number of actions performed to gain possession of the ball following an initial contest or deflection resulting from the distribution of the ball by the opposition.

Total Distance Covered (km)	Total distance covered.

Zone 4–Low Speed Sprinting (km)	Distance covered 19-23 km/h.

% Zone 4	Distance covered 19-23 km/h divided by the total distance.

% In Possession: Build Up Unopposed	Percentage of in possession time teams initiating the attacking play, with the aim of progressing the ball forwards up the pitch towards the opposition goal, under minimal pressure from the opponents.

% In Possession: Build Up Opposed	Percentage of in possession time initiating the attacking play, with the aim of progressing the ball forwards up the pitch towards the opposition goal, under extensive pressure from the opponents.

% In Possession: Progression	Percentage of in possession time advancing the ball during the attacking phase into the final third, achieved either through an initial vertical pass penetrating the opponent’s line or by a player an individual player dribbling the ball forward with a ball progression.

% In Possession: Final Third	Percentage of in possession time in the attacking third of the pitch, with the aim of scoring.

% In Possession: Long Ball	Percentage of in possession time of an attempted distribution actions (pass) of 25 yards or more is made.

% In Possession: Attacking Transition	Percentage of in possession time following the regaining possession of the ball, the team immediately attempts progress the ball forwards up the pitch towards the opposition goal with the intention of scoring a goal.

% In Possession: Counter-Attack	Percentage of in possession time regaining possession of the ball and immediately attacking the opposition.

% In Possession: Set Piece	Percentage of in possession time performing a corner, free-kick, throw-in and/or a penalty.

% Out of Possession: High-Press	Percentage of out of possession time applying defensive pressure against the opposition high up the pitch.

% Out of Possession: Mid-Press	Percentage of out of possession time applying defensive pressure against the opposition in the middle third of the pitch.

% Out of Possession: Low-Press	Percentage of out of possession time applying defensive pressure against the opposition in their defensive third of the pitch.

% Out of Possession: High-Block	Percentage of out of possession time adopting an organized defensive shape high up in the pitch.

% Out of Possession: Mid-Block	Percentage of out of possession time adopting an organized defensive shape in the middle third of the pitch.

% Out of Possession: Low-Block	Percentage of out of possession time adopting an organized defensive shape in their defensive third of the pitch.

% Out of Possession: Recovery	Percentage of out of possession time following the loss of the ball whereby a team returns towards their own goal in an attempt to defend their goal.

% Out of Possession: Defensive Transition	Percentage of out of possession time following the loss of ball possession, the team immediately attempts to defend progression of the opposition team toward their goal.

% Out of Possession: Counter-Press	Percentage of out of possession time following the loss of the ball, the team immediately attempts to regain possession of the ball.

Total Pressure	The total number of actions performed to close down the space on an opposition player in possession of the ball.

Direct Pressures	The total number of actions performed to close down space on an opposition player in possession of the ball that is close enough to the opposition play to be able to tackle/challenge/physically contest the ball and opposition action.

Average Pressure Duration (s)	The average duration of actions performed to close down the space on an opposition player in possession of the ball.

Ball Recovery Time (s)	The total time it takes to fully regain possession of the ball after losing possession.

Pushing on into Pressing	Total number of actions performed to close down space with an opposition player who does not have the ball and the action continues once the opposition player regains ball possession.

Pushing on	Total of actions performed to close down space with opposition player who does not have the ball.

Pressing Direction Inside	Total number of actions performed to directly and aggressively close down the space against and oppositon player to compete for ball possession inside of the opposition team shape.

Pressing Direction Outside	Total number of actions performed to directly and aggressively close down the space against and oppositon player to compete for ball possession outside of the opposition team shape.

TD (km/min)	Total distance (km) divided by the duration in minutes.

Z1 (km/min)	Distance between 0–7 km/h divided by the duration in minutes.

Z2 (km/min)	Distance between 7–13 km/h divided by the duration in minutes.

Z3 (km/min)	Distance between 13–19 km/h divided by the duration in minutes.

Z4 (km/min)	Distance between 19–23 km/h divided by the duration in minutes.

Z5 (km/min)	Distance above 23 km/h divided by the duration in minutes.

HSR (Z4; n/min)	Count of high-intensity runs divided by the duration in minutes.

SPR (Z5; n/min)	Count of sprinting runs divided by the duration in minutes.

Top Speed (km/h)	Top speed (maximum velocity).

The *key statistics* variables included possession (%), goals, attempts at goal, attempts at goal on target, percentage of attempts at goal on target, total passes, total passes completed, percentage of pass completion, line breaks completed, defensive line breaks, receptions in the final third, crosses, ball progressions, defensive pressures applied, direct pressures applied, percentage of direct pressures applied, forced turnovers, second balls, total distance covered (km), low speed sprinting (i.e., km covered in zone 4: 19–23 km/h) and percentage of low sprinting distance. The group of *in-possession* variables included the percentages per minute of build up unopposed, build up opposed, progression, final third, long ball, attacking transition, counter attack, and set piece, while the *out of possession* variables included the percentages per minute of high press, mid press, low press, high block, mid block, low block, recovery, defensive transition, and counter-press. The group of *out of possesion defensive presure* variables included total pressure, direct pressures, average pressure duration (s), forced turnovers, ball recovery time (s), pushing on into pressing, pushing on, pressing direction inside, and pressing direction outside. Finally, the group of physical performance variables consisted of total distance (TD in km), distance covered in zone 1 (0–7 km/h), distance covered in zone 2 (7–13 km/h), distance covered in zone 3 (13–19 km/h), distance covered in zone 4 (19–23 km/h), distance covered in zone 5 (> 23 km/h), number of high-speed runs (HSR – zone 4), number of sprints (SPR – zone 5), top speed (km/h).

### Statistical analysis

Linear mixed model analyses were conducted to analyze the match performance variables according to match outcome (result) and phase of the tournament (phase). Team and match identifiers were selected as random effects, and fixed effects included result and phase, analyzed as repeated measures, and their interaction. Normality of the residuals were assessed based on the Shapiro-Wilk normality test and visual inspection of the QQ-plots. Non-normally distributed data were log-transformed for the statistical analyses and backtransformed and presented as mean ± standard deviation. Where significant differences were observed, pairwise comparisons and the Bonferroni *post-hoc* analysis were conducted. Magnitude of significant differences were evaluated according to Cohen’s *d* effect size and interpreted based on the following criteria: <0.2, trivial, 0.2–0.6, moderate, 0.6–1.2, large, and > 2.0, very large [29].

## RESULTS

Table 2 summarizes the key statistics (data per minute) by match outcome (win, loss, and draw) and phase of the tournament (group or knock-out phase). In terms of match outcome, the results showed that there were significantly greater possession (%), goals, attempts at goal on target (%), total passes, total passes completed, defensive line breaks, and receptions in the final third when winning compared to drawing (small-to-moderate ES) or losing (moderate-to-large ES). Also, there were significantly greater completed line breaks when winning and drawing compared to losing (moderate ES) as well as significantly greater attempts at goal, crosses, ball progressions, and second balls when winning compared to losing (moderate ES). In addition, it was observed that teams that lost had significantly greater defensive pressures applied compared to teams that won or drew (moderate ES), and greater direct pressures than teams that won (moderate ES). Although the phase of the tournament had no significant effect on any variable (*p* > 0.05), significant interactions with moderate-to-very large ES were observed between match outcome and phase of the tournament in the following variables: attempts at goal on target (Groups: wins > losses and draws; Knock-out: wins > losses; Wins: groups > knock-out) and pass completion % (Groups: wins and draws > losses; Knock-out: wins > losses; Draw: groups > knock-out).

**TABLE 2:** Linear mixed models analyses for key statistics (data per minute): result (win, loss, draw) and phase of the tournament (group stage or knock-out phase).

Variables	Phase	Win	Loss	Draw	Main Effect		Match outcome × Phase (Interaction)	

					post-hoc	p	post-hoc	p
**%Possession**	**Groups**	48.05% ± 13.5% (28.1%)	37.19% ± 11.6% (31.1%) ***	42.93% ± 12.0% (28.0%)*	W > L (β), D (α)	<0.001	–	–

	**K/O**	51.98% ± 9.0% (17.3%)	36.18% ± 6.7% (18.6%) ***	41.91% ± 7.4% (17.6%) *				

**Goals**	**Groups**	0.027 ± 0.02 (65.3%)	0.009 ± 0 (5.8%) ***	0.013 ± 0 (38.9%) **	W > L (β), D (β)	<0.001	–	–

	**K/O**	0.024 ± 0.01 (49.9%)	0.01 ± 0 (4.2%) ***	0.011 ± 0 (47.1%) **				

**Attempts at Goal**	**Groups**	0.13 ± 0.05 (38.1%)	0.08 ± 0.04 (54.4%) ***	0.1 ± 0.05 (49.1%)	W > L (β)	<0.001	–	–

	**K/O**	0.12 ± 0.02 (19.6%)	0.09 ± 0.05 (53.1%) ***	0.12 ± 0.04 (36.5%)				

**Attempts at Goal – On Target**	**Groups**	0.06 ± 0.03 (47.5%)	0.02 ± 0.01 (64.8%) ***	0.02 ± 0.02 (68.2%) ***	-	-	**Grp:** W > L (†), D (†); **K/O**: W > L (β)	0.017

	**K/O**	0.04 ± 0.01 (26.8%)Δ	0.03 ± 0.01 (52.5%) ***	0.03 ± 0.02 (58.7%)				

**%Attempts at Goal – On Target**	**Groups**	40.77% ± 15.1% (37.1%)	23.69% ± 14.8% (62.3%) ***	26.21% ± 16.6% (63.4%) **	W > L (β), D (β)	<0.001	–	–

	**K/O**	36.91% ± 9.8% (26.6%)	36.94% ± 17.6% (47.6%) ***	39.92% ± 14.9% (31.2%) **				

**Total Passes**	**Groups**	4.57 ± 1.6 (34.0%)	3.31 ± 1 (29.7%) ***	4.07 ± 1.3 (31.2%) **	W > L (β), D (α)	<0.001	–	–

	**K/O**	5.25 ± 1.2 (22.9%)	3.68 ± 0.8 (21.5%) ***	3.69 ± 0.7 (20.0%) **				

**Total Passes** – Complete	**Groups**	3.72 ± 1.6 (43.9%)	2.41 ± 1 (40.5%)	3.14 ± 1.3 (41.5%)	W > L (β), D (α)	<0.001	–	–

	**K/O**	4.38 ± 1.2 (28.3%)	2.77 ± 0.8 (28.0%) ***	2.82 ± 0.7 (24.9%) **				

**% Pass Completion**	**Groups**	83.0% ± 5.5% (6.6%) ##	69.6% ± 9.7% (13.9%)	82.0% ± 2.4% (3.0%) ##	-	-	**Grp:** W, D > L (§); **K/O**: W > L (§); **D**: Grp > K/O	0.004

	**K/O**	82.4% ± 5.8% (7.0%) ##	74.2% ± 5.4% (7.3%)	77.1% ± 3.3% (4.3%)ΔΔ				

**Completed Line Breaks**	**Groups**	0.89 ± 0.3 (34.3%) ##	0.6 ± 0.2 (29.7%)	0.76 ± 0.3 (34.2%) #	W, D > L (β)	<0.001	–	–

	**K/O**	0.94 ± 0.2 (23.4%) ##	0.71 ± 0.2 (24.4%)	0.81 ± 0.2 (28.6%) #				

**Defensive Line Breaks**	**Groups**	0.1 ± 0.1 (51.5%)	0.04 ± 0 (69.5%) ***	0.06 ± 0 (52.5%) * ##	W > L (†), D (β);	<0.001	–	–

	**K/O**	0.09 ± 0 (42.7%)	0.06 ± 0 (49.8%) ***	0.08 ± 0 (31.0%) * ##	D > L (β)			

**Receptions in the Final Third**	**Groups**	1.23 ± 0.6 (46.2%)	0.69 ± 0.4 (53.7%) ***	0.88 ± 0.4 (42.1%) *	W > L (β), D (β)	<0.001	–	–

	**K/O**	1.27 ± 0.5 (36.0%)	0.84 ± 0.3 (33.8%) ***	1.04 ± 0.3 (28.8%) *				

**Crosses**		**Groups**	0.21 ± 0.1 (43.5%)	0.14 ± 0.1 (64.5%) **	0.17 ± 0.1 (56.6%)	W > L (β) 0.009	–	–

	**K/O**	0.17 ± 0.1 (43.3%)	0.15 ± 0.1 (43.0%) **	0.22 ± 0.1 (46.8%)				

**Ball Progressions**	**Groups**	0.21 ± 0.1 (38.8%)	0.16 ± 0.1 (41.7%) ***	0.19 ± 0.1 (29.9%)	W > L (β)	<0.001	–	–

	**K/O**	0.18 ± 0.1 (41.7%)	0.14 ± 0 (26.0%) ***	0.15 ± 0.1 (40.1%)				

**Defensive Pressures Applied**	**Groups**	1.86 ± 0.6 (29.8%) ##	2.5 ± 0.8 (31.0%)	2.17 ± 0.7 (30.3%) #	L > W (β), D (β)	<0.001	–	–

	**K/O**	1.77 ± 0.3 (19.2%) ##	2.62 ± 0.6 (22.1%)	2.04 ± 0.7 (32.1%) #				

**Defensive Pressures Applied – Direct Pressures**	**Groups**	0.53 ± 0.1 (27.6%) #	0.6 ± 0.2 (26.9%)	0.59 ± 0.1 (22.9%)	L > W (β)	0.014	–	–

	**K/O**	0.53 ± 0.1 (26.9%) #	0.67 ± 0.2 (24.2%)	0.61 ± 0.2 (35.3%)				

**%Defensive Pressures Applied (Direct Pressures)**	**Groups**	28.60% ± 6.1% (21.3%)	25.26% ± 6.3% (25.1%)	27.49% ± 4.4% (16.2%)	–	–	–	–

	**K/O**	29.78% ± 5.4% (18.0%)	26.16% ± 7.2% (27.6%)	30.07% ± 5.1% (16.9%)					

**Forced Turnovers**	**Groups**	0.79 ± 0.1 (17.2%)	0.78 ± 0.1 (14.2%)	0.79 ± 0.1 (17.0%)	–	–	–	–

	**K/O**	0.87 ± 0.1 (16.9%)	0.82 ± 0.1 (17.2%)	0.75 ± 0.1 (18.6%)				

**Second Balls**	**Groups**	0.94 ± 0.1 (13.7%)	0.84 ± 0.2 (21.6%) **	0.89 ± 0.1 (15.3%)	W > L (β)	0.005	–	–

	**K/O**	0.88 ± 0.1 (14.0%)	0.83 ± 0.1 (12.9%) **	0.85 ± 0.1 (17.1%)					

**Total Distance Covered (km)**	**Groups**	1.06 ± 0.1 (6.4%)	1.04 ± 0.1 (6.1%)	1.06 ± 0.1 (6.3%)	–	–	–	–

	**K/O**	1.08 ± 0.1 (6.6%)	1.07 ± 0.1 (7.3%)	1.04 ± 0 (4.0%)				

**Zone 4 – Low Speed Sprinting (km)**	**Groups**	0.05 ± 0 (12.3%)	0.05 ± 0 (13.9%)	0.05 ± 0 (13.1%)	–	–	–	–

	**K/O**	0.05 ± 0 (12.7%)	0.05 ± 0 (8.5%)	0.05 ± 0 (9.3%)				

**% Zone 4**	**Groups**	4.59% ± 0.5% (10.5%)	4.55% ± 0.5% (11.9%)	4.44% ± 0.6% (13.1%)	–	–	–	–

	**K/O**	4.48% ± 0.5% (11.5%)	4.43% ± 0.2% (4.3%)	4.66% ± 0.3% (5.7%)					

**Note**: Significantly different to W: * p <; 0.05; ** p <; 0.01; *** p <; 0.001. Significantly different to L: # p <; 0.05; ## p <; 0.01; ## p <; 0.001. Significantly different to D: $ p <; 0.05; $$ p <; 0.01; $$$ p <; 0.001. Significantly different to Group stages: Δ p <; 0.05; ΔΔ p <; 0.01; ΔΔΔ p <; 0.001. Cohen’s d effect size: α, small; β, moderate; †, large; §, very large. Data presented as mean ± SD (%CV).

Table 3 shows in possession and out of possession variables (data per minute) by match outcome (win, loss, and draw) and phase of the tournament (group stage or knock-out phase). Wins showed significantly greater build up unopposed and counter-press compared to losses and draws (moderate ES). Also, wins showed significantly greater high press (out of possession) and final third (%, in possession) than losses (moderate ES). Furthermore, mid press (out of possession) was greater in wins and draws compared to losses (moderate ES). However, there were significantly more long balls (in possession) and mid-block (%, out of possession) in losses than in wins (moderate ES) as well as significantly more counterattacks than draws (moderate ES). Losing and drawing teams had significantly more set pieces (small ES) and low block (%, out of possession, moderate ES) than winning teams. Although the phase of the tournament had no significant effect on any variable (*p* > 0.05), significant interactions (moderate ES) were observed between match outcome and phase of the tournament in the variable of progressions (Knockout: losses > wins).

**TABLE 3:** Linear mixed model analyses for in possession and out of possession variables (data per minute): result (win, loss, draw) and phase of the tournament (group stage or knock-out phase).

Possession	Variables	Phase	Win	Loss	Draw	Main Effect		Match outcome × Phase (Interaction)	

						post-hoc	p	post-hoc	p
**% IN POSSESSION**	**Build Up Unopposed**	**Groups**	29.87% ± 7.7% (25.8%)	22.11% ± 9.7% (43.8%) ***	26.75% ± 9.5% (35.4%) **	W > L (β), D (β)	<0.001	–	–

		**K/O**	33.27% ± 6.1% (18.2%)	22.17% ± 8.7% (39.1%) ***	23.50% ± 5.0% (21.5%) **				

	**Build Up Opposed**	**Groups**	11.32% ± 4.1% (36.5%)	10.03% ± 3.4% (33.8%)	11.95% ± 4.5% (37.8%)	–	–	–	–

		**K/O**	12.25% ± 3.2% (25.8%)	10.50% ± 2.8% (26.5%)	10.25% ± 2.9% (28.4%)				

	**Progression**	**Groups**	15.24% ± 2.7% (18.0%)	14.42% ± 2.6% (18.2%)	14.40% ± 2.9% (19.8%)	–	–	**K/O**: L > W (β)	0.013

		**K/O**	14.25% ± 1.4% (10.0%) #	16.08% ± 2.5% (15.3%)Δ	15.14% ± 2.0% (12.9%)				

	**Final Third**	**Groups**	24.51% ± 6.5% (26.3%)	17.71% ± 6.7% (37.7%) ***	20.00% ± 6.9% (34.3%)	W > L (β)	<0.001	–	–

		**K/O**	22.42% ± 5.4% (24.2%)	20.42% ± 6.0% (29.5%) ***	24.88% ± 5.0% (20.2%)				

	**Long Ball**	**Groups**	1.71% ± 1.4% (82.3%) ##	2.82% ± 1.5% (53.4%)	2.45% ± 2.1% (84.2%)	L >W (β)	<0.001	–	–

		**K/O**	1.83% ± 1.1% (60.8%) ##	3.25% ± 1.5% (45.7%)	3.13% ± 1.6% (49.7%)				

	**Attacking Transition**	**Groups**	21.44% ± 6.2% (28.8%)	23.63% ± 6.6% (28.0%)	20.80% ± 5.5% (26.6%)	–	–	–	–

		**K/O**	17.83% ± 5.7% (31.7%)	22.42% ± 7.2% (32.0%)	20.75% ± 2.8% (13.6%)				

	**Counter Attack**	**Groups**	1.53% ± 1.0% (62.3%)	1.76% ± 1.1% (60.6%)	1.18% ± 0.7% (61.8%) ##	L > D (β)	0.011	–	–

		**K/O**	1.25% ± 0.8% (60.3%)	1.91% ± 0.9% (49.4%)	1.25% ± 0.5% (37.0%) ##				

	**Set Piece**	**Groups**	8.22% ± 2.4% (29.7%) ## $	9.14% ± 2.3% (25.6%)	9.10% ± 3.2% (35.3%) L, D >	W (α)	0.004	–	–

		**K/O**	6.82% ± 1.8% (26.9%) ## $	9.92% ± 2.5% (25.2%)	10.50% ± 3.0% (28.3%)				

**% OUT OF POSSESSION - (Rotate text check)**	**High Press**	**Groups**	4.08% ± 1.8% (44.3%)	2.76% ± 1.6% (58.9%) **	2.95% ± 2.0% (69.1%)	W > L (β)	0.006	–	–

		**K/O**	3.55% ± 1.4% (40.6%)	2.92% ± 1.5% (51.6%) **	3.50% ± 1.5% (43.2%)				

	**Mid Press**	**Groups**	5.95% ± 2.0% (33.4%) ##	4.63% ± 1.6% (35.3%)	6.50% ± 2.1% (32.9%) ##	W, D > L (β)	<0.001	–	–

		**K/O**	6.42% ± 2.5% (39.0%) ##	5.17% ± 1.3% (25.9%)	6.00% ± 2.2% (36.7%) ##				

	**Low Press**	**Groups**	0.87% ± 0.6% (71.7%)	1.26% ± 0.7% (54.2%)	0.85% ± 0.4% (43.1%)	–	–	–	–

		**K/O**	1.00% ± 0.6% (60.3%)	0.92% ± 0.7% (72.9%)	1.13% ± 0.6% (57.0%)				

	**High Block**	**Groups**	5.32% ± 3.1% (58.2%)	4.05% ± 2.6% (63.4%)	4.47% ± 3.0% (66.4%)	–	–	–	–

		**K/O**	4.92% ± 2.1% (42.0%)	3.70% ± 2.2% (58.5%)	5.29% ± 1.5% (28.3%)				

	**Mid Block**	**Groups**	14.72% ± 6.6% (45.1%) #	18.11% ± 7.7% (42.3%)	16.20% ± 4.5% (28.0%)	L > W (β)	0.018	–	–

		**K/O**	14.82% ± 5.3% (35.7%) #	22.27% ± 7.7% (34.6%)	15.63% ± 4.4% (28.4%)				

	**Low Block**	**Groups**	9.17% ± 4.7% (51.2%) ## $$$	16.67% ± 9.3% (56.1%)	15.80% ± 9.0% (56.8%)	L, D > W (β)	<.001	–	–

		**K/O**	13.92% ± 6.3% (45.4%) ## $$$	17.00% ± 6.8% (40.2%)	15.75% ± 4.7% (29.5%)				

	**Recovery**	**Groups**	4.21% ± 2.1% (49.4%)	4.05% ± 1.7% (41.7%)	4.00% ± 1.5% (38.2%)	–	–	–	–

		**K/O**	4.20% ± 1.5% (35.1%)	3.92% ± 1.6% (41.4%)	3.86% ± 1.1% (27.7%)				

	**Defensive Transition**	**Groups**	23.63% ± 6.6% (28.0%)	21.73% ± 6.0% (27.8%)	20.80% ± 5.5% (26.6%)	–	–	–	–

		**K/O**	22.50% ± 7.1% (31.6%)	17.83% ± 5.7% (31.7%)	20.75% ± 2.8% (13.6%)				

	**Counter-press**	**Groups**	16.92% ± 4.6% (27.5%)	14.45% ± 4.2% (28.9%) ***	15.10% ± 3.9% (26.0%) *	W > L (β)	0.006	–	–

		**K/O**	16.25% ± 5.0% (31.0%)	12.75% ± 3.6% (28.6%) ***	14.13% ± 2.2% (15.3%) *				

**Note**: Significantly different to W: * p <; 0.05; ** p <; 0.01; *** p <; 0.001. Significantly different to L: # p <; 0.05; ## p <; 0.01; ## p <; 0.001. Significantly different to D: $ p <; 0.05; $$ p <; 0.01; $$$ p <; 0.001. Significantly different to Groups stage: Δ p <; 0.05; ΔΔ p <; 0.01; ΔΔΔ p <; 0.001. Cohen’s d effect size: α, small; β, moderate; †, large; §, very large. Data presented as mean ± SD (%CV).

Table 4 displays out of possession defensive pressure variables (data per minute) by match outcome (win, loss, and draw) and phase of the tournament (group stage or knock-out phase). Teams that lost showed significantly greater total pressure, pressing direction outside, and ball recovery time compared to teams that won or drew (moderate ES). Also, teams that lost showed significantly greater direct pressures, pushing on into pressing, pushing on, pressing direction inside compared to teams that won (moderate ES). The phase of the tournament had no significant effect on any of these out of possession defensive pressure variables (*p* > 0.05). However, significant interactions (moderate-to-very large ES) were observed between match outcome and phase of the tournament in the variable of average pressure duration (Groups: draws > wins; Knock-out: losses > wins > draws; Losses: knock-out > groups; Draws: Groups > knock-out).

**TABLE 4:** Linear mixed model analyses for out of possession defensive pressure variables (data per minute): result (win, loss, draw) and phase of the tournament (group stage or knock-out phase).

Variables	Phase	Win	Loss	Draw	Main Effect		Match outcome × Phase (Interaction)	

					post-hoc	p	post-hoc	p
**Total Pressure**	**Groups**	1.86 ± 0.6 (29.8%) ##	2.5 ± 0.8 (31.0%)	2.17 ± 0.7 (30.3%) #	L > W (β), D (β)	<0.001	–	–

	**K/O**	1.77 ± 0.3 (19.2%) ##	2.62 ± 0.6 (22.1%)	2.04 ± 0.7 (32.1%) #				

**Direct Pressures**	**Groups**	0.53 ± 0.1 (27.6%) #	0.6 ± 0.2 (26.9%)	0.59 ± 0.1 (22.9%)	L > W (β)	0.014	–	–

	**K/O**	0.53 ± 0.1 (26.9%) #	0.67 ± 0.2 (24.2%)	0.61 ± 0.2 (35.3%)				

**Avg Pressure Duration (s)**	**Groups**	0.014 ± 0 (9.5%) $	0.014 ± 0 (6.3%)	0.015 ± 0 (8.3%)	–	–	Grp: D > W (β); **K/O**: L > W (β) > D (§);	<0.001
	
	**K/O**	0.014 ± 0 (9.4%) #	0.015 ± 0 (7.2%) ΔΔ	0.012 ± 0 (13.7%) * ## ΔΔΔ			L: **K/O** > Grp (†); D: Grp > **K/O** (§)	

**Forced Turnovers**	**Groups**	0.79 ± 0.1 (17.2%)	0.78 ± 0.1 (14.2%)	0.79 ± 0.1 (17.0%)	–	–	–	–

	**K/O**	0.87 ± 0.1 (16.9%)	0.82 ± 0.1 (17.2%)	0.75 ± 0.1 (18.6%)				

**Ball Recovery Time (s)**	**Groups**	0.082 ± 0 (21.2%) ##	0.107 ± 0 (29.6%)	0.09 ± 0 (25.7%) ##	L > W, D (β)	<0.001	–	–

	**K/O**	0.079 ± 0 (14.5%) ## Δ	0.106 ± 0 (21.2%) Δ	0.068 ± 0 (16.9%) ## Δ				

**Pushing on into Pressing**	**Groups**	0.71 ± 0.3 (35.5%) ##	0.98 ± 0.4 (41.2%)	0.82 ± 0.3 (39.6%)	L > W (β)	<0.001	–	–

	**K/O**	0.63 ± 0.2 (33.2%) ##	0.91 ± 0.3 (32.0%)	0.7 ± 0.3 (40.8%)				

**Pushing on**	**Groups**	1.63 ± 0.5 (32.3%) ##	2.22 ± 0.8 (37.9%)	1.85 ± 0.7 (36.7%)	L > W (β)	<0.001	–	–

**K/O**	1.51 ± 0.4 (26.4%) ##	2 ± 0.6 (28.3%)	1.61 ± 0.5 (30.9%)					

**Pressing Direction Inside**	**Groups**	0.29 ± 0.1 (29.8%) ##	0.36 ± 0.1 (34.4%)	0.32 ± 0.1 (32.8%)	L > W (β)	<0.001	–	–

	**K/O**	0.29 ± 0.1 (19.6%) ##	0.42 ± 0.1 (24.8%)	0.32 ± 0.1 (42.3%)				

**Pressing Direction Outside**	**Groups**	0.92 ± 0.3 (37.5%) ##	1.34 ± 0.5 (36.2%)	1.11 ± 0.4 (34.2%) #	L > W, D (β)	<0.001	–	–

	**K/O**	0.91 ± 0.2 (26.3%) ##	1.39 ± 0.4 (27.3%)	1.01 ± 0.3 (34.2%) #				

Note: Significantly different to W: * p <; 0.05; ** p <; 0.01; *** p <; 0.001. Significantly different to L: # p <; 0.05; ## p <; 0.01; ## p <; 0.001. Significantly different to D: $ p <; 0.05; $$ p <; 0.01; $$$ p <; 0.001. Significantly different to **Groups** stage: Δ p <; 0.05; ΔΔ p <; 0.01; ΔΔΔ p <; 0.001. Cohen’s d effect size: α, small; β, moderate; †, large; §, very large. Data presented as mean ± SD (%CV).

Table 5 shows the physical performance variables (data per minute) by match outcome (win, loss, and draw) and phase of the tournament (group stage or knock-out phase). According to match outcome, there were no significant differences in any variable, except for distance covered in zone 1 (distance covered in losses > distance covered in draws, moderate ES). The phase of the tournament had no significant effect on any physical performance variable, except for distance covered in zone 1 (distance covered in knock-out > distance covered in groups). There were no significant interactions between match outcome and phase of the tournament in the physical performance variables.

**TABLE 5:** Linear mixed model analysis of relative (data per minute) team physical performance: result (win, loss, draw) and phase of the tournament (group stage or knock-out phase).

Variables	Phase	Win	Loss	Draw	Main Effect		Match outcome × Phase (Interaction)	

					post-hoc	p	post-hoc	p
**TD (km/min)**	**Groups**	1.06 ± 0.1 (6.4%)	1.04 ± 0.1 (6.1%)	1.06 ± 0.1 (6.3%)	–	–	–	–

	**K/O**	1.08 ± 0.1 (6.6%)	1.07 ± 0.1 (7.3%)	1.04 ± 0 (4.0%)				

**Z1 (km/min)**	**Groups**	0.38 ± 0 (4.7%)	0.37 ± 0 (4.8%) $$	0.39 ± 0 (4.3%)	D > L (β)	<0.001	–	–

	**K/O**	0.39 ± 0 (3.8%) ΔΔΔ	0.39 ± 0 (2.3%) $$ ΔΔΔ	0.4 ± 0 (2.8%) ΔΔΔ	**K/O** > Grp (β)	<0.001		

**Z2 (km/min)**	**Groups**	0.39 ± 0 (12.2%)	0.37 ± 0 (12.5%)	0.38 ± 0 (11.5%)	–	–	–	–

	**K/O**	0.39 ± 0.1 (14.4%)	0.39 ± 0.1 (14.4%)	0.35 ± 0 (8.3%)				

**Z3 (km/min)**	**Groups**	0.23 ± 0 (9.9%)	0.23 ± 0 (8.8%)	0.23 ± 0 (10.1%)	–	–	–	–

	**K/O**	0.24 ± 0 (6.5%)	0.24 ± 0 (7.8%)	0.22 ± 0 (8.9%)				

**Z4 (km/min)**	**Groups**	0.049 ± 0.006 (12.3%)	0.047 ± 0.007 (13.9%)	0.047 ± 0.006 (12.9%)	–	–	–	–

	**K/O**	0.048 ± 0.006 (12.8%)	0.047 ± 0.004 (8.9%)	0.049 ± 0.005 (9.6%)				

**Z5 (km/min)**	**Groups**	0.019 ± 0.004 (20.6%)	0.018 ± 0.004 (23.8%)	0.018 ± 0.004 (20.7%)	–	–	–	–

	**K/O**	0.019 ± 0.004 (23.1%)	0.019 ± 0.004 (20.2%)	0.019 ± 0.003 (15.1%)				

**HSR (Z4; n/min)**	**Groups**	19.02 ± 1.8 (9.5%)	18.76 ± 1.6 (8.8%)	18.92 ± 1.6 (8.7%)	–	–	–	–

	**K/O**	19.03 ± 1.8 (9.6%)	18.89 ± 2 (10.4%)	18.12 ± 1.8 (9.8%)				

**SPR (Z5; n/min)**	**Groups**	4.25 ± 0.6 (13.0%)	4.12 ± 0.5 (12.4%)	4.07 ± 0.4 (9.7%)	–	–	–	–

	**K/O**	4.21 ± 0.5 (11.3%)	4.13 ± 0.2 (6.1%)	4.18 ± 0.3 (7.9%)				

**Top speed (km/h)**	**Groups**	31.41 ± 0.9 (2.9%)	31.18 ± 1.1 (3.4%)	31.15 ± 0.9 (3.0%)	–	–	–	–

	**K/O**	31.15 ± 0.9 (2.8%)	31.53 ± 1.1 (3.5%)	32.16 ± 1 (3.2%)				

Note: Significantly different to W: * p <; 0.05; ** p <; 0.01; *** p <; 0.001. Significantly different to L: # p <; 0.05; ## p <; 0.01; ### p <; 0.001. Significantly different to D: $ p <; 0.05; $$ p <; 0.01; $$$ p <; 0.001. Significantly different to **Groups** stage: Δ p <; 0.05; ΔΔ p <; 0.01; ΔΔΔ p <; 0.001. Cohen’s d effect size: α, small; β, moderate; †, large; §, very large. Data presented as mean ± SD (%CV).

## DISCUSSION

The purpose of this study was to analyze the FIFA 2023 Women’s World Cup match performance according to match outcome and phase of the tournament. Significant differences were observed in key statistics (e.g., there were significantly greater possession, goals, attempts at goal on target, total passes, total passes completed, defensive line breaks, and receptions in the final third when winning compared to drawing or losing), in possession variables (e.g., wins showed significantly greater build up unopposed compared to losses and draws), out of possession variables (e.g., wins showed significantly greater high press than losses), and out of possession defensive pressure variables (e.g., losses showed significantly greater total pressure, pressing direction outside, and ball recovery time compared to wins or draws). However, there were no significant differences in any physical performance variable, except for distance covered in zone 1 (distance covered in losses > distance covered in draws). For the phase of the tournament, no significant differences were observed in any variable, except for distance covered in zone 1 (distance covered in knock-out > distance covered in groups). Furthermore, significant interactions were observed between match outcome and phase of the tournament in the following variables: attempts at goal on target, pass completion, progressions, and average pressure duration.

The results of this study are consistent with a previous study analyzing the technical performance of soccer teams at the FIFA 2019 Women’s World Cup [12]. For example, Kubayi & Larkin (2020) indicated that winning teams demonstrated significantly greater performance compared to losing teams across variables such as total passes, passing accuracy, ball possession, shots, shots on target, ball recovery patterns, aerial challenges, and set piece indicators. With regards to creating shots, it is worth mentioning that data from the EURO 2022 tournament revealed that creating shots did not only depend on possession length but also on the rate of passes [30]. This may be also linked to variables like visual exploratory activity (e.g., higher scan frequencies have been particularly noted in central defensive midfield positions, where players were more likely to turn with the ball and successfully maintain possession rather than lose it) [31]. On the other hand, Kubayi & Larkin (2020) indicated that losing teams lost the ball more often, registered more tackles, and received more yellow cards compared to winning teams. In addition, one of the novel findings of our study was the key role of out of possession variables (e.g., wins showed significantly greater high press than losses) and out of possession defensive pressure variables (e.g., losses showed significantly greater total pressure, pressing direction outside, and ball recovery time compared to wins or draws). These findings are thought-provoking, especially given previous research that has concluded possession regains high up the field are crucial to a team’s attacking success [13, 32].

In terms of physical performance, this study found no significant differences in any variable, except for distance covered in zone 1 (distance covered in losses > distance covered in draws). However, data from the last FIFA Women’s World Cup (2023) suggest that increasing the capacity of the team to cover distance at high speed might be beneficial to achieving success in the elite level [33]. A previous study analyzed physical output in matches with 90-minute duration (home and away matches from the regular season) and concluded that, in general, match demands were not influenced by factors such as match outcome (win vs loss) [34]. However, differences in physical and technical characteristics have been noted both between and within age groups, depending on match status and possession status, which suggests that teams adjust their playing style according to match status (likely to influence or maintain the scoreline) [35]. There is conflicting evidence regarding the impact of match outcome on physical demands not only in soccer but also in a variety of field sports [34]. A recent study in male professional soccer players observed that match outcome had a relation to variables such as distance covered and HSR [36]. Also, another study found significant correlations between points obtained at the end of the league and variables such as distance covered with ball possession (r – 0.75), distance covered without ball possession (r – -0.70), sprinting actions with ball possession (r – 0.55), sprinting distance with ball possession (r – 0.49), and maximum speed (r – 0.41) [37]. In addition, the main findings were that high ranked teams covered the greatest total distance with ball possession, sprinting distance with ball possession, and completed the greatest number of sprinting actions with ball possession and maximal velocity [38]. Caution should be taken when interpreting this data because most of the research available to date comes from male professional soccer players in the context of regular league seasons, indicating a need for more research in female soccer players.

Finally, no significant differences were observed in key statistics, in possession and out of possession variables for the phase of the tournament. Only significant differences were found in distance covered in zone 1 (distance covered in knock-out > distance covered in groups). To the best of the authors’ knowledge, this is one of the first studies analyzing multiple teams’ performances between different phases of a tournament such as the FIFA Women’s World Cup. Previous research has aimed to identify the performance variables that best differentiate between eliminated and qualified teams for the knockout phase of different tournaments (e.g., World Cup or Champions League) [23, 39, 40]. For instance, a previous study from the FIFA 2018 Men’s World Cup showed that winning teams had more ball possession and pass success rates during the group stage of the tournament, and they spent more time at high intensities as well as achieving more shots and shots on target independent of whether the match was performed in the group or final knockout stage [40]. The fact that distance covered in zone 1 (low speed) in the knock-out phase was greater than the distance covered in the group phase of the 2023 Women’s World Cup suggests that the distribution of match intensity may change as teams progress to the knock-out stage.

This study presents several limitations which need to be acknowledged. For example, the nature of this tournament requires considering matches which are significantly longer than the standard 90-minute match because of 30-minute extra-time, which may be considered as a limitation since matches with different durations were included. Also, grouping match observations solely by match outcome can be an overly simplistic method that fails to capture the dynamic nature of match status during observations, potentially resulting in inaccurate categorization [8]. For instance, a team might score in the final minutes of a match after being tied for most of the game, yet still be classified as a win [8]. Future research should also investigate the impact of evolving match status (i.e., drawing, winning, or losing) on performance [8].

## CONCLUSIONS

Quantifying and comprehending match-play characteristics hold significant importance in guiding practices within women’s soccer. This study represents an initial effort to consolidate scientific literature assessing the match-play traits of women’s soccer, encapsulating the physical, technical, and tactical aspects across the group phase and knock-out stage of the 2023 FIFA Women’s World Cup. Therefore, this study serves as a resource for the staff of national teams to understand match performance variables related to success in an international women’s soccer tournament.

The study has highlighted that significant differences in match outcome are observed in specific variables related to technical-tactical performance. These differences may be considered by the teams’ staff to design their training strategies, which may include small-sided games and other possession-based exercises, specific attacking patterns, breaking defensive lines, effective final third movements, improving decision making in goal-scoring areas, and train players on how to break through opposition’s defensive lines; however, teams need to know how to limit opponent’s success in these same areas. Another suggestion is the implementation of defensive drills that enhance players’ ability to recover the ball quickly and reduce recovery time. Also, it may be recommended to incorporate high-intensity training drills that focus on pressing immediately after losing possession, particularly in the final third. Nonetheless, training when to implement a high press versus a mid-press, adjusting their strategies based on the phase of play and opposition’s weaknesses might be another practical application. These are, among others, a few examples of how to use some of the findings in training settings, but experts on technical/tactical development and teams’ staff could adapt these training strategies considering the findings of the study and their specific aims, styles of play, and perspectives of each team. In this regard, the use of match footage would be necessary to highlight successful implementations and areas needing improvement.
